# Cytokine profile in the sputum of subjects with post-tuberculosis airflow obstruction and in those with tobacco related chronic obstructive pulmonary disease

**DOI:** 10.1186/s12865-020-00381-w

**Published:** 2020-10-01

**Authors:** Elise Guiedem, Eric Walter Pefura-Yone, George Mondinde Ikomey, Céline Nguefeu Nkenfou, Martha Mesembe, Mbanyamsig Mispa Yivala, Bih Hycenta Chendi, Graeme Brendon Jacobs, Novel Njweipi Chegou, Marie Claire Assoumou Okomo

**Affiliations:** 1 Center for the Study and Control of Communicable Diseases (CSCCD), Faculty of Medicine and Biomedical Sciences, University of Yaoundé 1, Yaoundé, Cameroon; 2 Pneumological Service, Yaounde Jamot Hospital, Yaoundé, Cameroon; 3 Chantal BIYA International Reference Centre for Research on HIV/AIDS Prevention and Management (CBIRC), Yaoundé, Cameroon; 4 Higher Teachers Training College, University of Yaoundé 1, Yaoundé, Cameroon; 5 DST-NRF Centre of Excellence for Biomedical Tuberculosis Research; and SAMRC Centre for Tuberculosis Research, Division of Molecular Biology and Human Genetics, Department of Biomedical Sciences, Faculty of Medicine and Health Sciences, Stellenbosch University, PO Box 241, Cape Town, 8000 South Africa; 6 Division of Medical Virology, Department of Pathology, Faculty of Medicine and Health Sciences, Stellenbosch University, PO Box 241, Cape Town, 8000 South Africa

**Keywords:** Bronchial obstruction, Cells, COPD, Cytokines, Inflammation, Tobacco, Tuberculosis

## Abstract

**Background:**

Previous studies have shown that tuberculosis (TB) is a risk factor for chronic airflow limitation. Chronic obstructive pulmonary disease (COPD) is recognized as the result of chronic inflammation, usually related to noxious particles. Post-TB airflow obstruction and tobacco-related COPD have the same functional pathway characterized by persistent airflow limitation. We sought to compare the profile of 29 cytokines in the sputum of subjects with post-TB airflow obstruction and those with COPD related to tobacco.

**Results:**

The forced expiratory volume in the first second (FEV1) and forced expiratory volume/forced vital capacity (FEV/FVC) ratio were lower in the COPD patients with the history of smoking compared to the post-TB airflow obstruction subgroup. The stages of the disease were more advanced in COPD / tobacco patients.

Among the cytokines, IL-1α, IL-1β, MIP-1β, sCD40L and VEGF levels were higher in COPD patients, compared to the controls with *p* values ​​of 0.003, 0.0001, 0.03, 0.0001 and 0.02 respectively. When the two COPD subgroups were compared, IL-1α, IL-6, TNF-α and IL-8 levels were higher in the COPD patients with the history of tobacco compared to the COPD patients with the history of TB with *p*-values ​​of 0.031, 0.05, 0.021 and 0.016, respectively.

**Conclusion:**

COPD related to tobacco is more severe than post-TB airflow obstruction. The pathogenesis of post-TB airflow obstruction appears to involve the cytokines IL-1RA, IL-1α, IL-1β, IL-17, GRO and sCD40L, while COPD related to tobacco involves more cytokines.

## Background

Chronic obstructive pulmonary disease (COPD) is a respiratory disease characterized by progressive airflow obstruction that is not fully reversible, with combined emphysema and chronic obstructive bronchitis [1]. It is a major cause of morbidity and mortality worldwide and is gradually increasing. It can lead to abnormal inflammation in the airways. The diagnosis of COPD is based mainly on clinical symptomatology and measurement of spirometric variables. Clinical symptomatology include: evocative signs of chronic bronchial diseases (recurring bronchial episodes, wheezes) and chronic respiratory signs (cough, expectoration, effort dyspnea) [1]. The spirometric diagnosis of COPD include the forced expiratory volume in the first second (FEV1), the forced vital capacity (FVC) and FEV/FVC ration. COPD is defined as the FEV/FVC ratio less than 70%, or the lower limit of normal, after inhalation of 400 μg of salbutamol and the absence of complete reversibility. The severity of the COPD is evaluated according to four clinical stages: Stage I (mild) with FEV1 ≥ 80%, Stage II (moderate) with 50% ≤ FEV1 < 80%, Stage III (severe) with 30% ≤ FEV1 < 50% and Stage IV (very severe) with FEV1 < 30% [2].

Cigarette smoking is known as the main risk factor of COPD [2]. The defense system of the respiratory airways includes the mucociliary carpet, which cleans respiratory airways, and the epithelial tight junctions, which form a physical barrier between tissues and airway space. This barrier can be broken by chronic exposure to cigarette smoke, which irritates the lung wall by damaging epithelial cells and tissue [3]. TNF-α produced by Alveolar macrophages and epithelial cells, stimulates the influx of neutrophiles and others monocytes/macrophages to the airway tract. Macrophages and epithelial cells will secrete cytokines such as IL-8, GRO, MCP-1 and MIP-1 α [4]. These cytokines have several actions in the physiopathology of COPD and then subsequently amplify the inflammatory process during disease [3, 4].

The damage induced by cigarette smoke leads to the limitation of respiratory flow that induces emphysema and chronic bronchitis that are peculiar to COPD.

Although smoking is the main cause of COPD [5], the same functional syndromes, characterized by persistent airflow obstruction, are increasingly observed in patients with previous pulmonary tuberculosis (TB) who are non smokers [6]. Some studies have shown that TB is a risk factor for COPD, with COPD observed in approximately 7.6% of patients who previously had TB [6, 7]. Other studies have identified post-TB airflow obstruction in patients who have a similar syndrome, with a history of pulmonary TB and have never smoked.

Post-TB airflow limitation is also common after TB treatment [8]. Inflammation of the bronchial endothelium leads to localized or generalized bronchial obstruction, hypertrophy of the submucous glands and smooth muscles, pulmonary fibrosis and edema of airways mucosa, with increased secretion of mucus [9]. The results of these events increase airway resistance to airflow [10]. The destruction of the parenchyma affects lung compliance by increasing the tendency of small pathways to collapse, the main characteristic of COPD [11].

Knowledge of the inflammatory characteristics of COPD and/or post-TB AFO could help to better adapt the anti-inflammatory treatment of patients, and thus reduce the frequent phenomenon of corticoresistance [12]. The purpose of this study was to determine the profile of cytokines in COPD patients, with a history of smoking compared to those with a history of TB.

## Results

### Demographic characteristics

A total of 150 participants were recruited: 90 COPD patients and 60 clinically healthy non-smokers for the control group. The COPD patients consisted of 50 patients with a history of smoking (COPD/tobacco) and 40 patients who previously had TB (COPD/post-TB) (Table 1). A significant difference was observed between COPD patients with a history of smoking and the patients with post-TB airflow obstruction with regards to sex and age, with *p* values of 0.0023 and 0.0002, respectively. A multivariate analyzes indicated that spirometric data were not influenced by the sex and age, with the following results: for the sex, odd ratio (IC 95%) = 2.0 (1.05–10.71) and *p* = 0.54; for the age, odd ratio = 2.74 (1.15–109.3) and *p* value of 0.51.

**Table 1:** Demographic and spirometric data of all participants

	COPD/post-TB	COPD/tobacco	Control	p
Age	40 ± 2.1	63 ± 10.45	43 ± 12.38	0.0002
Male	16 (40%)	42 (84%)	40 (66.7%)	0.002
Female	24 (60%)	8 (16%)	20 (33.3%)	
FEV1	53.30% (± 17.21%)	36.88% (± 14.95%)	83 ± 6.56%	< 0.0001
FVC	84.89 ± 15,1%	72.97 ± 14.5%	97.64 ± 4.15%	< 0.0001
FEV1/FVC	62.79% (± 17.95%)	50.54% (± 12.09%).	85 ± 6.06%	< 0.0001
Stage of disease	Stage I: 0	Stage I: 0		0.038
	Stage II: 20 (50%)	Stage II: 11 (22%)		
	Stage III: 15 (37.5%)	Stage III: 14 (28%)		
	Stage IV: 5 (12.5%)	Stage IV: 25 (50%)		

*FEV1* Forced expiratory volume in the first second, *FVC* Forced vital capacity, *FEV1/FVC* Forced expiratory volume/ forced vital capacity ratio

### Spirometric characteristics

In the COPD patients with a smoking history, FEV1 ranged from 20.3 to 64.6% and in the post-TB airflow obstruction patients, FEV1 ranged from 30.0 to 79.0%. In the control group, FEV/FVC ranged from 70.0 to 97%. The FEV/FVC ratio of COPD/tobacco patients ranged from 35.0 to 68.0% and in the post-TB airflow obstruction patients, it ranged from 35.0 to 74.4%. The FEV1 and FEV/FVC ratio was lower in the COPD patients with a history of tobacco than in the post-TB airflow obstruction patients with *p*-values ​​of 0.014 and 0.033, respectively. As a result, the stage of COPD was more advanced in COPD patients with a smoking history compared to COPD patients with anterior TB with a *p*-value of 0.032 (Table 1).

### Concentrations of cytokines in participants

The detected cytokines in the sputum were anti-cytokines (IL-1RA), pro-inflammatory cytokines (IL-1α, IL-1β, IL-6, IL-17 and TNF-α), chemokines (MCP-1, IL-8, MIP-1α, MIP-1β, GRO, IP-10 and sCD40L) and growth factors (VEGF, G-CSF and GM-CSF). Other cytokines such as IFN-α, IFN-γ, IL-10, IL-12, MDC, PDGF, IL-15, IL-2, IL-4, IL-7 and RANTES were not detected in the sputum of either the COPD patients or the control group.

Table 2 shows the mean (± SD) concentrations of cytokines in sputum in the three groups: in patients with COPD, the levels of cytokines such as IL-1RA, IL-1α, IL-1β, MIP-1β, sCD40L and VEGF were statistically higher compared to the control group with *p*-values ​​all lower at 0.05. IL-6 and TNF-α were higher in COPD patients with a history of tobacco compared to the post-TB airflow obstruction patients and to the control. GRO concentration was higher in COPD patients with anterior TB than in the control. There were no statistically significant differences in cytokine concentrations, such as IL-17, GM-CSF, G-CSF, MIP-1α and IP-10.

**Table 2:** Sputum cytokine concentrations in patients and controls

Cytokines	COPD/post-TB	COPD/tobacco	Control	p
Anti-cytokines (pg/mL)				
IL-1RA	1945 (±873.1)	1720 (±491.1)	637.7 ± 191.3	0.046
Pro-inflammatory cytokines (pg/mL)				
IL-1 α	821.2 ± 205.7	1555 ± 360.7	539.1 ± 198.6	0.003
IL-1β	137.9 ± 65.25	296.4 ± 143.5	9.410 ± 3.781	< 0.0001
IL-6	9.966 ± 6.916	16.23 ± 7.768	11.64 ± 2.90	0.04
IL-17A	2.412 ± 0.41	1.54 ± 0.2650	1.421 ± 0.23	0.141
TNF-α	1.110 ± 0.5649	7.112 ± 4.542	1.880 ± 0.31	0.0095
Chemokines (pg/mL)				
MCP-1	254.3 ± 101.4	657.8 ± 276.3	522.7 ± 69.00	0.08
IL-8	103.0 ± 68.58	493.1 ± 244.9	135.5 ± 30.93	0.023
MIP-1 α	11.12 ± 6.6	10.20 ± 4.9	23.64 ± 6.2	0.1674
MIP-1β	224.1 ± 103.4	181.1 ± 63.57	81.65 ± 20.27	0.03
GRO	14,047 ± 13,020	1721 s ± 977.7	2563 ± 1617	0.05
IP-10	420.8 ± 239.5	242.2 ± 123.9	848.4 ± 314.0	0.069
sCD40L	1.958 ± 0.384	1.506 ± 0.441	0.286 ± 0.164	< 0.0001
Growth factors (pg/mL)				
VEGF	333.9 ± 136.5	435.2 ± 130.2	332 ± 155.8	0.02
G-CSF	237.1 ± 146.4	592.3 ± 227	774.3 ± 207.9	0.68
GM-CSF	1.920 ± 0.35	592.3 ± 400.6	1.55 ± 0.35	0.34

Meaning of abbreviations: *pg/mL* Picogram per milliliter, *IL* Interleukin, *IL-1RA* IL-1 receptor antagonist, *TNF* Tumor necrosis factor, *GRO* Growth regulated oncogene, *G-CSF* Ganulocyte-Colony Stimulating Factor, *GM-CSF* Granunocyte Macrophage- CSF, *IP* Interferon-inductible protein, *MIP* Macrophage inflammatory protein, *MCP* Monocyte chemotactic protein, *sCD40L* Soluble cluster differentiation 40 ligand, *VEGF* Vascular endothelium growth factor

**Fig. 1:**
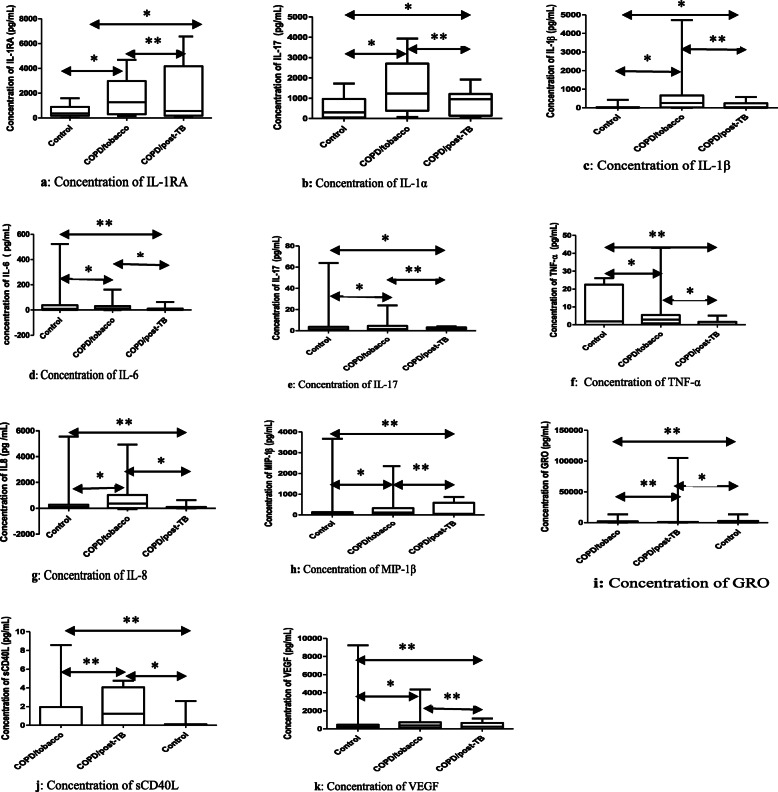
Variation of cytokines and comparison of their concentrations between two groups. **a**: Concentration of IL-1RA. **b**: Concentration of IL-1α. **c**: Concentration of IL-1β. **d**: Concentration of IL-6. **e**: Concentration of IL-17. **f**: Concentration of TNF-α. **g**: Concentration of IL-8. **h**: Concentration of MIP-1β. **i**: Concentration of GRO. **j**: Concentration of sCD40L. **k**: Concentration of VEGF. Legend: : Comparison between 2 groups. *: significant difference. **: non significant difference. IL: interleukin. IL-1RA: IL-1receptor antagonist. GRO: growth regulated oncogene. TNF: tumor necrosis factor. MIP: macrophage inflammatory protein. MCP: monocyte chemiotactic protein. sCD40L: soluble cluster differentiation 40 ligand. VEGF: vascular endothelial growth factor. - The **Significant difference** means that the concentration of the cytokine between the two groups compared is statistically different with a *p* value less than or equal to 0.05. - The **Non significant difference** means that the concentration of cytokine between the two groups compared is not statistically different with a p value greater than 0.05

Figure 1 show the variation of cytokines and the comparison of their concentration using the Student’s T-test or Mann-Whitney U test. When the two subgroups of COPD patients were compared with each other, the levels of IL-1α, IL-6, TNF-α and IL-8 were higher in the COPD patients with a tobacco history than in the patients with post-TB airflow obstruction with *p*-values of 0.031; 0.049; 0.021 and 0.016 respectively, with no significant differences on the other cytokines (Fig. 1).

The comparison of COPD patients with the history of smoking to the control group indicated that in COPD/tobacco patients, the levels of cytokines, such as IL-1RA, IL-1α, IL-1β, IL-6, TNF-α, IL-17, IL − 8 MIP-1β and VEGF, were statistically higher compared to the control group, with *p*-values of 0.014; 0.006; 0.025; 0.033; 0.048, 0.041; 0.009, 0.012 and 0.035 respectively (Fig. 1), without statistically significant differences in the concentrations of cytokines, such as IP-10, MCP-1, GRO, sCD40L, G-CSF and GM-CSF (Fig. 1).

The comparison of COPD patients with the history of tuberculosis with the control group showed that the concentrations of six cytokines, IL-1RA, IL-1β, IL-1α, IL-17, GRO and sCD40L were higher in patients with the history of tuberculosis with *p*-values of 0.014; 0.048; 0.029; 0.045; 0.028 and 0.0013, respectively (Fig. 1), without statistically significant difference in the levels of the other cytokines, such as G-CSF, GM-CSF, MIP-1α, MIP-1β, MCP-1, IL-6, TNF-α, IP-10, IL-8, and VEGF between the control group and COPD with anterior TB (Fig. 1).

### Correlation between clinical stage, cells and cytokine levels in sputum

**Fig. 2:**
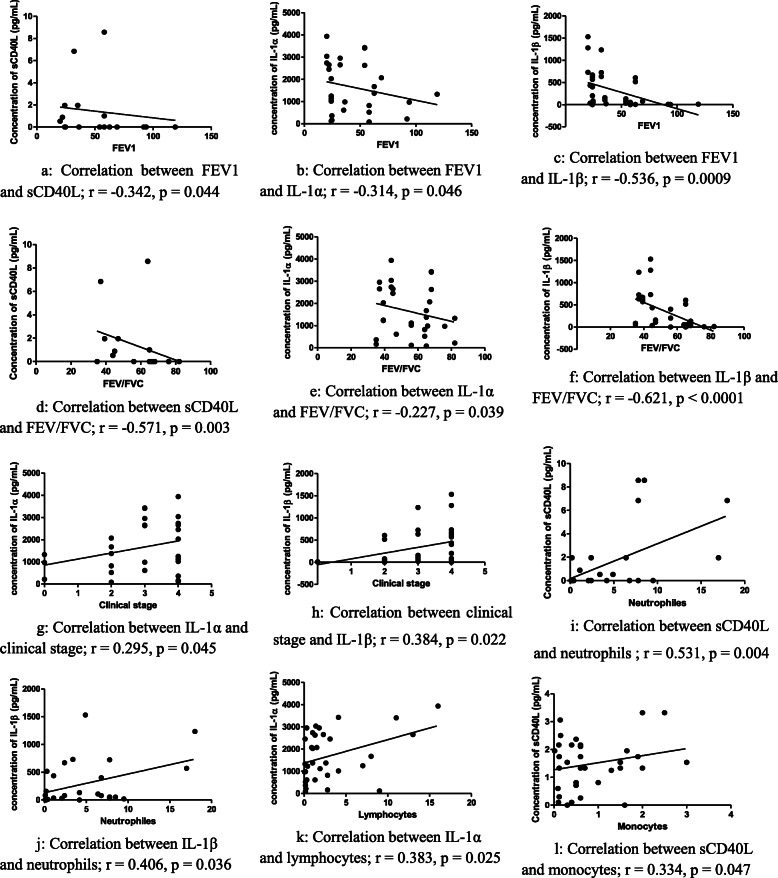
Significant correlations between cytokines and spirometric data or cells in the COPD patients with the history of smoking. **a**: Correlation between FEV1 and sCD40L; *r* = -0.342, *p* = 0.044. **b**: Correlation between FEV1 and IL-1α; *r* = -0.314, *p* = 0.046. **c**: Correlation between FEV1 and IL-1β; *r* = -0.536, *p* = 0.0009. **d**: Correlation between sCD40L and FEV/FVC; *r* = -0.571, *p* = 0.003. **e**: Correlation between IL-1α and FEV/FVC; *r* = -0.227, *p* = 0.039. **f**: Correlation between IL-1β and FEV/FVC; *r* = -0.621, *p* < 0.0001. **g**: Correlation between IL-1α and clinical stage; *r* = 0.295, *p* = 0.045. **h**: Correlation between clinical stage and IL-1β; *r* = 0.384, *p* = 0.022. **i**: Correlation between sCD40L and neutrophils ; *r* = 0.531, *p* = 0.004. **j**: Correlation between IL-1β and neutrophils; *r* = 0.406, *p* = 0.036. **k**: Correlation between IL-1α and lymphocytes; *r* = 0.383, *p* = 0.025. **l**: Correlation between sCD40L and monocytes; *r* = 0.334, *p* = 0.047. Legend: FEV1: forced expiratory volume in the first second. FVC: forced vital capacity. IL: interleukin. sCD40L: soluble cluster differentiation 40 ligand. *r* = Coefficient of correlation: - When **r** is positive, this means that the concentration of cytokine and the values of spirometric data (or the concentration of cells) vary in the same direction. - When **r** is negative, this means that the concentration of cytokine and the value of spirometric data (or concentration of cells) vary in the opposite direction. *p* = significance threshold: - If **p** is greater than 0.05, the correlation is not significantly different. - If **p** is less than or equal to 0.05, the correlation is statistically significant

Figure 2 shows the significant correlations between cytokines and spirometric data or cells in the COPD patients with a history of smoking. The significant correlations were: The lower the FEV1 was, the higher the concentrations of sCD40L, IL-1α and IL-1β were (Fig. 2). The lower the FEV/FVC ratio was, the higher the concentrations of sCD40L, IL-1α and IL-1β were (Fig. 2). The more advanced the clinical stage was, the higher the IL-1α and IL-1β concentrations were, with correlation coefficients of 0.295 and 0.384 respectively and *p*-values ​​below 0.05 (Fig. 2). Statistically significant positive correlations were found between neutrophils levels and cytokines IL-1β and sCD40L, between lymphocytes and IL-1α and between monocytes and sCD40L (Fig. 2). The non significant correlations were: There were negative non significant correlations between FEV1 and cytokines IL-17, IL-6, MIP-1α, MIP-1β and IL-8. There were negative non significant correlations between FEV/FVC ratio and cytokines IL-17, MIP-1α and IL-8. There were positive non significant correlations between clinical stage and cytokines GRO, sCD40L, IL-17, IL-6, MIP-1β and IL-8. The positive non significant correlations were found between neutrophils and IL-1α, between lymphocyte and cytokines such as sCD40L, IL-6, MIP-1β and TNF-α and between monocytes and cytokines such as IL-17, IL-1β, IL-1α and IL-8.

**Fig. 3:**
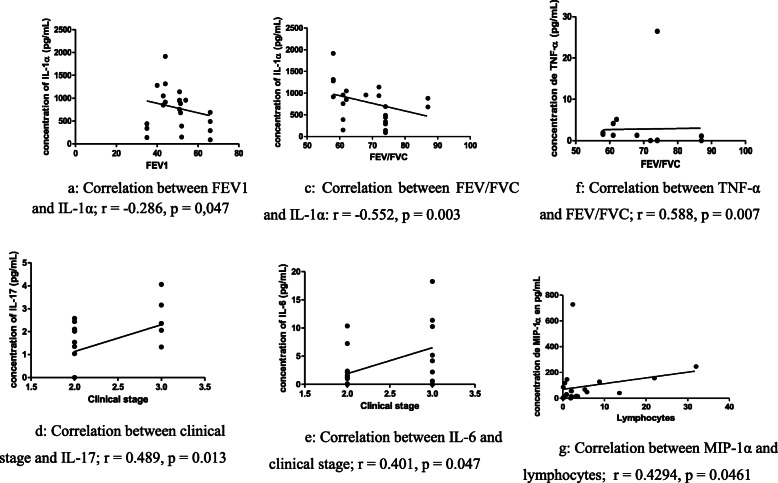
Significant correlations between cytokines and spirometric data or cells in the post-TB airflow obstruction patients. **a**: Correlation between FEV1 and IL-1α; *r* = -0.286, *p* = 0,047. **c**: Correlation between FEV/FVC and IL-1α: *r* = -0.552, *p* = 0.003. **d**: Correlation between clinical stage and IL-17; *r* = 0.489, *p* = 0.013. **e**: Correlation between IL-6 and clinical stage; *r* = 0.401, *p* = 0.047. **f**: Correlation between TNF-α and FEV/FVC; *r* = 0.588, *p* = 0.007. **g**: Correlation between MIP-1α and lymphocytes; *r* = 0.4294, *p* = 0.0461. Legend: FEV1 = forced expiratory volume in the first second. FVC = forced vital capacity. IL = interleukin. MIP = macrophage inflammatory protein. *r* = correlation test. *r* = Coefficient of correlation: - When **r** is positive, this means that the concentrations of cytokine and the values of spirometric data (or the concentrations of cells) vary in the same direction. - When **r** is negative, this means that the concentrations of cytokine and the value of spirometric data (or concentrations of cells) vary in the opposite direction. *p* = significance threshold: - If **p** is greater than 0.05, the correlation is not significantly different. - If **p** is less than or equal to 0.05, the correlation is statistically significant

Figure 3 shows the significant correlations between cytokines and spirometric data or cells in the patients with post-TB airflow obstruction: The significant correlations were: The lower the FEV1 was, the higher the concentration of IL-1α was (Fig. 3). The lower the FEV/FVC ratio was, the higher the concentrations of IL-1α and TNF-α were (Fig. 3). The more advanced the clinical stage was, the higher the IL-17 and IL-6 concentrations were, with correlation coefficient of 0.489 and 0.401 with *p*-values ​​of 0.013 and 0.047. Statistically significant positive correlation was found between lymphocytes levels and MIP-1α (Fig. 3: Fig. 3g). The non significant correlations were: There were negative non significant correlations between FEV1 and cytokines such as IL-17, IL-1β, IL-6, MIP-1α, MIP-1β, TNF-α and IL-8. There were negative non significant correlations between FEV/FVC ratio and cytokines such as GRO, sCD40L, IL-1β, IL-6, MIP-1α MIP-1β and IL-8. There were positive non significant correlations between clinical stage and cytokines such as: GRO, sCD40L, IL-1β, IL-1α, MIP-1α, MIP-1β, TNF-α and IL-8. The positive non significant correlations were found: between neutrophils and cytokines sCD40L, IL-1β and MIP-1β, between lymphocyte and cytokines IL-17, IL-1α, IL-6, MIP-1α, MIP-1β, TNF-α and IL-8 and between monocytes and cytokines sCD40L, IL-1β, IL-1α, IL-6, TNF-α and IL-8.

## Discussion

We analyzed the spirometric characteristics, cell profile and cytokine profile in COPD patients with a history of tobacco, with a history of previous TB and in healthy participants who served as a control group. This is significant and relevant because we observed important differences.

Concerning spirometric characteristics, FEV1 and FEV/FVC were lower in the COPD patients with a smoking history than in the COPD patients with post-TB airflow obstruction. COPD patients with a history of tobacco were at a more advanced stage of the disease. These results suggest that the disturbances and irritations caused by smoking are more pronounced than those induced by TB. In addition, the treatment of TB eliminates mycobacterial infection and the damage associated with the mycobacterium itself is reduced, while in smokers, smoking continues to cause damage until the development of COPD [13]. Moreover, the duration of smoking and the time being treated for TB may also induce this difference of severity between sub-groups of COPD patients.

Cytokines of the IL-1 family intervene in the inflammatory process [14]; IL-1α, IL-1β and IL-1RA were assayed. Levels of IL-1α were statistically higher in the COPD patients with a history of smoking compared to post-TB subgroup. The study found through association tests that IL-1α was strongly correlated with the clinical stage of the disease. Since the stage of COPD was more advanced in the COPD subgroup with a history of smoking, this could justify the greater presence of IL-1α in smokers compared to patients with post-TB airflow obstruction. IL-1RA, IL-1α and IL-1β were higher in the COPD patients with a smoking history and post-TB airflow obstruction subgroups, in comparison to the control group, with statistically significant differences. The results for post smoking COPD were similar to those found by Fernando et al. [14] that showed a strong IL-1β expression following exposure to tobacco smoke. In a study by Rusznak et al. [13], they noted significant secretion of IL-1β by epithelial cells cultured from COPD patients with a history of smoking. IL-1RA (IL-1 receptor antagonist) is a cytokine inhibitor present in all cells that express IL-1, more particularly by monocytes/macrophages, and has an anti-inflammatory role [15]. Since there is bronchial hyperactivity during COPD, IL-1RA is increased in patients, most likely with the aim of reducing the effect of high concentrations of IL-1α and IL-1β [16, 17] at the origin of this hyperactivity, hence the high concentrations in COPD patients who previously had TB and in tobacco related COPD. The function of IL-1α was previously investigated in mice, which indicated a high level of IL-1α 4 days after exposure to cigarette smoke, during stable COPD and during exacerbations [18]. IL-1α may promote neutrophils infiltration into the airway. The study performed by Fernando et al. (2011) showed a significant correlation between IL-1α and IL-1β levels and chemokine in the sputum of mice with COPD. IL-1α and IL-1β could therefore promote the production of other pro-inflammatory cytokines.

The higher IL-6 in COPD patients with a history of smoking compared to COPD patients who previously had TB could be related to two facts: either IL-6 does not significantly interfere with post-TB airflow obstruction (there was no difference between the COPD/post-TB subgroup and the control group in the level of IL-6), or this result could be linked to the fact that most COPD/post-TB subjects were at a less advanced clinical stage (the results showed a positive correlation between the clinical stage and the concentration of IL-6). Suleyman et al. [19] found that the clinical stage of COPD was correlated with the concentration of IL-6.

Levels of TNF-α were statistically significantly higher in the COPD patients with a history of smoking. Generally, the secretion of TNF-α in COPD is induced by tobacco smoke and maintained by the chronic inflammation process [20]. TNF-α has multiple pro inflammatory actions [21]. TNF-α stimulates the migration of monocytes/macrophages and neutrophils into the epithelial airways. Macrophages and induced epithelial cells produce GRO, MIP-1 and IL-8 [22]. TNF-α and IL-8 cause degranulation of neutrophils and shortness of breath with the production of free radicals that causes epithelial and matrix damage. In addition, TNF-α has also been reported to have a direct effect on epithelial cells [22], being capable of inducing hypersecretion of mucus, resulting in cell death and emphysematous lesions and contributing to the deterioration of the clinical state seen in COPD correlated with weight loss [3].

Since *Mycobacterium tuberculosis* is no longer present in the post-TB airflow obstruction patients, there is no real inducing element of TNF-α, which could justify its low concentration in COPD/post-TB patients.

IL-17 concentrations in both subgroups of COPD patients were higher compared to the control group. During post smoking COPD, IL-17 plays a role in T cell proliferation, activation of fibroblasts, endothelials and epithelial cells, induces release of cytokines (IL-6, GM-CSF) and activation of neutrophils [23, 24]. IL-17 is involved in the proliferation of T lymphocytes, activation of fibroblasts, endothelial and epithelial cells. IL-17 induces the release of Il-6 and IL-8 [25]. The fact that the IL-17 concentration is high in the COPD/post TB subgroup could be justified by the fact that this cytokine also plays an important role in post-TB AFO as in COPD related to tobacco.

Regarding MIP-1β, a higher concentration was noted in the COPD patients with a history of tobacco compared to the control group. MIP-1β is a chemokine that induces lymphocyte migration and recognizes CCR5 receptors in T cells and macrophages [26]. Some studies have shown that MIP-1β is essentially produced by macrophages [26]. Our results indicated a higher concentration among the smokers certainly because these patients were at a more advanced stage of the disease (the lesions caused by the destruction of the airways were more severe), and this could be related to emphysema that characterizes COPD. MIP-1β plays an important role in the IgG immune complex induced by acute lung injury [27]. This chemokine was not strongly expressed in the post-TB airflow obstruction patients. This could be explained by the fact that in this subgroup most patients were at a less advanced stage of the disease; so emphysematous damage should be lower compared to smokers.

The high expression of IL-8 in smoker patients has certainly been induced by cigarette smoke, and is maintained by chronic intoxication of the airways as is the case in the studies conducted by Becker and colleagues [28]. Studies report that myeloperoxidase and elastase released by activated neutrophils amplify the production of IL-8 [29], which would justify its high concentration in COPD/tobacco patients who have a higher number of neutrophils. Our recent study on the same population showed that the neutrophil count was higher in post-smoking COPD compared to the post-TB form [30]. GRO is an important chemotactic mediator of neutrophils, endothelial cell adhesion and degranulation. Its chemotactic role in neutrophils has been demonstrated by some studies based on the animal model [29]. It would play the same role as IL-8 in tobacco related COPD. The concentration of GRO was rather high in the post-TB airflow obstruction patients (post-TB AFO). Since this chemokine is high in the sputum of patients with a history of TB, it could be the chemotactic marker of neutrophils in post-TB AFO such as IL-8 in the post smoking COPD form. However, the preferential involvement of the GRO in post-TB AFO remains to be clarified.

SCD40L bind to CD40 of monocytes to promote their adhesion to the vascular endothelium, and to endothelial cells to activate them. Some studies have found high concentrations of sCD40L in certain diseases such as severe sepsis [31] and in coronary syndrome [32], but, the studies has yet not shown the role of sCD40L in COPD neither in humans nor in the animal model. It is possible that sCD40L is an important marker in COPD and could play a particular role in the pathogenesis of post-TB airflow obstruction through its high concentration.

VEGF was strongly expressed in the sputum of COPD patients with a smoking history compared to the control group, and not expressed in the COPD patients with anterior TB. VEGF is a growth factor involved in angiogenesis (forming new preexisting vessel growths) in asthma. As in asthma, VEGF would be involved in the mechanism of bronchial vascular remodeling during COPD. Kanazawa et al. [33] found an increased level of VEGF in the induced sputum of subjects with COPD. Kranenburg et al. [34] showed that COPD was associated with increased VEGF expression in the bronchi and bronchial and alveolar epithelium. Two studies conducted by Sichelstiel et al. [35] and Esmaeil et al. [16] found higher concentrations of VEGF in the sputum of COPD patients compared to healthy subjects. Calabrese et al., [27] found an association between increased bronchial vasculature and cellular expression of VEGF and that VEGF receptor blockade induced apoptosis of alveolar endothelial cells. The high pulmonary concentrations of VEGF in COPD, and more particularly in smokers, may therefore reflect two effects: either a regulatory effect upstream of the irritations caused by tobacco smoke, or an attempt to repair epithelial damage related to pathogenesis and emphysema in COPD [33].

## Conclusion

The objective of the study was to compare the profile of certain cytokines in COPD/tobacco and COPD/post-TB patients. We reached the following conclusions: COPD related to tobacco use is more severe than the post-TB form. The pathogenesis of COPD in patients with a history of smoking involves several cytokines in the local pathway such as IL-1α, IL-1β, IL-6, IL-17, TNF-α, IL-8, MIP-1β and VEGF. In contrast the pathogenesis of the post-TB form seems to imply fewer inflammatory markers with IL-1RA, IL-1β, GRO and sCD40L as local marker.

## Methods

### Study participants

Study participants were recruited from the Yaoundé Jamot Hospital (YJH) from February 2016 to July 2017. YJH is the main and biggest center in Cameroon specializing in the management of respiratory disorders. Participants constituted COPD patients and healthy non-smokers who served as controls. COPD participants were chosen among patients consulting at the YJH and who had been diagnosed by a pneumologist. COPD patients comprised two sub-groups: patients with a history of smoking (COPD/tobacco) and patients with post-TB airflow obstruction (post-TB AFO or COPD/post TB). The COPD/tobacco group had clinical signs of COPD during active smoking and they stopped to smoke once they understood that cigarette smoke was the cause of their disease. Post-TB AFO patients had previously one or more episode TB without smoking. Participants were eligible for the study if they gave written informed consent for participation, including consent for HIV testing. Patients were excluded if they had active TB, or were physically or mentally unable to perform a respiratory function test. The work received ethical clearance from the Cameroon National Ethical Committee of Research for Human Health (N° 2016/06/772/CE/CNERSH/SP).

### Data and sample collection

Once the pneumologist had diagnosed an eligible patient with COPD, the spirometric results of the patient were extracted from his/her medical record. The history of TB or smoking was also extracted from patients’medical records and confirmed by each patient. Spirometric measurements for the control group were done with the turbine pneumotachograph (SpiroUSB, Care fusion, Yorba Linda, USA) following the American Thoracic Society standard to ensure that participants did not have any respiratory problems. Sputum samples were collected from each participant. Samples were transported at ambient conditions in a coolbox to the Center for the Study and Control of Communicable Diseases of the Faculty of Medicine and Biomedical Sciences, University of Yaoundé I. Upon receipt in the laboratory, cell counts (neutrophils, macrophages and lymphocytes) were performed after staining of the smear with May-Grunwald Giemsa and read by light microscopy. After the cell counts, the sputum was liquefied by mixing with phosphate buffered saline (PBS). The sputum was centrifuged at 2000 rpm for 10 min, and the supernatant was aliquoted and stored at − 80 °C until cytokine analyses. The cytokine analyses were performed by the Luminex technique at Stellenbosch University Immunology Research Group laboratory (SUN-IRG), university of Stellenbosch, Cape Town, South Africa.

### Luminex multiplex immunoassay

The concentrations of 29 cytokines including pro-inflammatory cytokines (interleukin: IL-1β, IL-1α, IL-2, IL-4, IL-6, IL-7α, IL-12p70, IL-12p40, IL-15 and IL-17A, tumor necrosis factor: TNF-α and TNF-β, and interferon: IFN-α and IFN-ɣ), chemokines (IL-8, growth regulated oncogene: GRO, interferon-inductible protein: IP-10, macrophage inflammatory protein: MIP-1α and MIP-1β, monocyte chemiotactic protein: MCP-1, regulated on activation normal T cells expressed and secreted: RANTES, soluble CD40 ligand: sCD40L and macrophage-derived chemokine: MDC); anti-cytokines (IL-10 and IL-1receptor antagonist: RA) and growth factors (ganulocyte-colony stimulating Factor: G-CSF, granunocyte macrophage-CSF: GM-CSF, platelet-derived growth factor: PDGF and vascular endothelial growth factor: VEGF), were investigated in sputum samples from all study participants. Reagents were purchased from Merck Millipore, Billerica, Massachusetts, United States of America (USA). Experiments were performed and read on the Bio-Plex platform (Bio-Rad, USA), with the Bio-Plex Manager Software version 6.1 used for bead acquisition and analysis.

### Statistical analyses

The data was analyzed using Graphpad Prism version 5 (GraphPad Software Inc., California, USA). Quantitative variables were presented as mean (± standard error) when the distribution was considered normal; if not they were represented by their median (± interquartile interval). A chi-square test was used to compare proportions. The Student’s t-test or Mann-Whitney U test was used to compare means or medians respectively between two groups. The ANOVA test was used to compare the quantitative variables between three groups at the same time. The Pearson’s chi squared test was used to test the association between the concentrations of cytokines and spirometric data. *P*-values ≤0.05 were considered significant.

## Data Availability

The datasets used and/or analysed during the current study available from the corresponding author on reasonable request.

## Abbreviations

AFO: Airflow obstruction
ATS: American Thoracic Society
CD: Cluster differentiation
CI: Confidence interval
COPD: Chronic obstructive pulmonary disease
CSCCD: Center for the Study and Control of Communicable Diseases
FEV: Forced expiratory volume in the first second
FVC: Forced vital capacity
G-CSF: Ganulocyte-colony stimulating Factor
GM-CSF: Granunocyte macrophage-CSF
GOLD: Global initiative for obstructive lung diseases
IFN: Interferon
Ig: Immunoglobuline
IL: Interleukin
IL-1RA: IL-1receptor antagonist
RA IP: Interferon-inducible protein
MCP: Monocyte chemotactic protein
MDC: Macrophage-derived chemokine
MGG: May–Grunwald Giemsa
MIG: Monokine induced by interferon-gamma
ml: Milliliter
MMP: Metalloproteinase matrix
PDGF: Platelet-derived growth factor
RANTES: Regulated on activation normal T cells expressed and secreted
sCD40L: Soluble CD40 ligand
TB: Tuberculosis
TNF: Tumor necrosis factor
VEGF: Vascular endothelial growth factor
YJH: Yaoundé Jamot Hospital
